# Diverse Virus and Host-Dependent Mechanisms Influence the Systemic and Intrahepatic Immune Responses in the Woodchuck Model of Hepatitis B

**DOI:** 10.3389/fimmu.2020.00853

**Published:** 2020-05-27

**Authors:** Tomasz I. Michalak

**Affiliations:** Molecular Virology and Hepatology Research Group, Division of BioMedical Sciences, Faculty of Medicine, Health Sciences Centre, Memorial University of Newfoundland, St. John's, NL, Canada

**Keywords:** woodchuck model of hepatitis B, virus-hepatocyte interaction, major histocompatibility complex presentation, asialoglycoprotein receptor, hepatocyte as cytotoxic immune effector, intrahepatic innate and adaptive immune responses, pre-acute infection, toll-like receptors

## Abstract

Woodchuck infected with woodchuck hepatitis virus (WHV) represents the pathogenically nearest model of hepatitis B and associated hepatocellular carcinoma (HCC). This naturally occurring animal model also is highly valuable for development and preclinical evaluation of new anti-HBV agents and immunotherapies against chronic hepatitis (CH) B and HCC. Studies in this system uncovered a number of molecular and immunological processes which contribute or likely contribute to the immunopathogenesis of liver disease and modulation of the systemic and intrahepatic innate and adaptive immune responses during hepadnaviral infection. Among them, inhibition of presentation of the class I major histocompatibility complex on chronically infected hepatocytes and a role of WHV envelope proteins in this process, as well as augmented hepatocyte cytotoxicity mediated by constitutively expressed components of CD95 (Fas) ligand- and perforin-dependent pathways, capable of eliminating cells brought to contact with hepatocyte surface, including activated T lymphocytes, were uncovered. Other findings pointed to a role of autoimmune response against hepatocyte asialoglycoprotein receptor in augmenting severity of liver damage in hepadnaviral CH. It was also documented that WHV in the first few hours activates intrahepatic innate immunity that transiently decreases hepatic virus load. However, this activation is not translated in a timely manner to induction of virus-specific T cell response which appears to be hindered by defective activation of antigen presenting cells and presentation of viral epitopes to T cells. The early WHV infection also induces generalized polyclonal activation of T cells that precedes emergence of virus-specific T lymphocyte reactivity. The combination of these mechanisms hinder recognition of virus allowing its dissemination in the initial, asymptomatic stages of infection before adaptive cellular response became apparent. This review will highlight a range of diverse mechanisms uncovered in the woodchuck model which affect effectiveness of the anti-viral systemic and intrahepatic immune responses, and modify liver disease outcomes. Further exploration of these and other mechanisms, either already discovered or yet unknown, and their interactions should bring more comprehensive understanding of HBV pathogenesis and help to identify novel targets for therapeutic and preventive interventions. The woodchuck model is uniquely positioned to further contribute to these advances.

## Introduction

Woodchuck hepatitis virus (WHV) was discovered in wild-caught woodchucks housed at the Philadelphia Zoological Garden (Pennsylvania, USA) where chronic hepatitis (CH) and hepatocellular carcinoma (HCC) were observed at high rates (1, 2). Studies that followed demonstrated that the virus is highly compatible to HBV considering ultrastructure, genome organization and size, nucleotide sequence (~65% homology), and in replication strategy. The number and functions of viral proteins and the range of organs targeted, namely the liver and the immune system, were also alike (3–7). WHV and HBV also share similar profiles of endurance of viral antigens in circulation and liver, and liver disease displays the stepwise progression where acute hepatitis (AH) spontaneously resolves and is followed by persistent occult infection or advances to CH and HCC (2, 5, 8–13). The profiles of WHV-specific humoral and cell-mediated, as well as innate immune responses closely model those in human hepatitis B. Resolution of AH coincides with vigorous, polyclonal T cell response, intrahepatic upregulation of interferon (IFN)-γ and IFN-α, and appearance of otherwise protective antibodies against WHV envelope (surface) antigen (WHsAg). In contrast, CH is characterized by weak or absent T cell reactivity toward virus, T cell exhaustion, and a decreased hepatic expression of interferons (14–19)]. Importantly, both infections comparably respond to antiviral and to the majority of immunomodulatory approaches tested so far, and the pharmacokinetic and drug toxicity are congruent (12, 20–22). For these reasons, naturally or experimentally infected eastern North American woodchucks (*Marmota monax*) have been recognized as the pathogenically and immunologically relevant model of human HBV infection and HBV-induced liver diseases, and as the preferable system for assessing potential future therapeutics. Nevertheless, there are certain differences between HBV and WHV and liver diseases caused. On the molecular level, there are discrepancies in the activities of individual enhancers and promoters (23) and differences in glycosylation of the virus envelope proteins (24). Considering liver pathology, the leading variances are that CH does not progress to cirrhosis and that HCC advances at much higher rate (80–90%) in chronically infected woodchucks than in CH patients who acquired infection in adulthood in whose HCC rate is considerably lower (~5%) (8, 10, 11).

This review summarizes studies according to their focus but not based on the time line when the data were reported. Also, the review does not cover the entire scope of contributions made by the woodchuck model, particularly not those where the model was applied for evaluation of antiviral and immunomodulatory therapies since such summaries were recently published by others (12, 25). The purpose of this review was to bring together the range of the mechanisms uncovered, on one hand, to illustrate their vast diversity and, on the other, to encourage broader exploration of this highly valuable model to advance our knowledge beyond the scope explored so far. This should expedite discovery of new therapeutic and preventive strategies against both virus and diseases which it causes.

## Types of WHV Infection and Stages of WHV-Initiated Liver Disease

Approximately 250 million people have serum HBV surface antigen (HBsAg)-reactive CH and up to two billion may have occult HBV infection occurring in the absence of detectable HBsAg and clinically apparent hepatic disease (26). Symptomatic infection usually begins as serum HBsAg-positive AH which subsides without treatment in the majority of adults and, therefore, was named as self-limited AH (SLAH). SLAH is followed by asymptomatic low-level persistence of HBV, termed as secondary occult infection (SOI) or seropositive occult infection (OBI) (27, 28). This infection continues in the absence of serum HBsAg but is accompanied by antibodies to HBV core (anti-HBc) antigen and to HBsAg (anti-HBs), and by low levels of circulating HBV DNA, while virus replication is readily detectable in the liver and the immune system. Up to one-tenth of patients with AH advances to CH which is serum HBsAg and anti-HBc reactive disease usually accompanied by high levels of circulating HBV DNA of up to 10^10^-10^11^ virus genome copies, also called virus genome equivalents (vge), per mL. CH displays biochemical and histological indicators of liver protracted inflammation and hepatocyte death, robust HBV replication, and it may progress to liver cirrhosis and HCC, which are the main causes of mortality among chronically infected patients.

**Figure 1:**
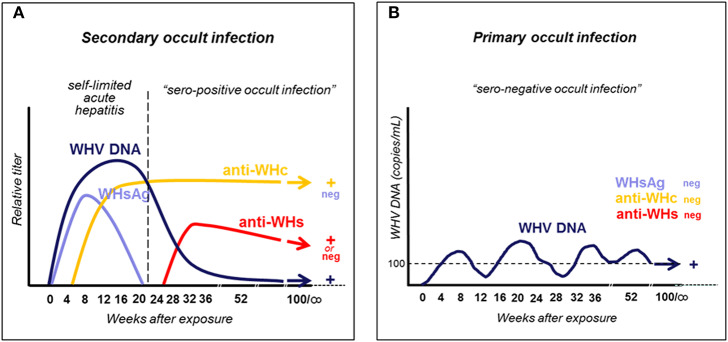
Schematic presentation of serum immunovirological and molecular profiles of occult infections caused by WHV. **(A)** Secondary occult infection (SOI) continuing after resolution of self-limited acute hepatitis caused by a liver pathogenic dose greater or equal to 1 × 10^3^ WHV characterized by persistent asymptomatic infection negative for serum WHsAg but reactive for anti-WHc antibodies, positive or negative for anti-WHs antibodies, and positive for WHV DNA at levels below 100–200 virus copies/mL. **(B)** Primary occult infection (POI) developing after exposure to a liver non-pathogenic dose of <1 × 10^3^ WHV virions that persists as a seronegative infection but remains serum WHV DNA reactive at levels usually below 100–200 virus copies/mL.

Woodchucks naturally or experimentally infected with WHV develop AH which is seropositive for WHsAg and antibodies to WHV core (nucleocapsid) antigen (anti-WHc), and is accompanied by biochemical and histological signs of liver injury. Like in HBV-infected humans, AH can advance to CH and HCC or may resolve and lifelong SOI is established (Figure 1A) (5, 13, 29). Resolution of AH coincides with apparent complete clearance of serum WHsAg (see section below), arise of antibodies to WHsAg (anti-WHs), decrease in serum WHV DNA to levels below 100–200 vge/mL, and normalization of liver biochemical function. Notably, like in HBV-infected patients, CH is diagnosed when WHsAg reactivity in plasma or serum persists for longer than 6 months. In addition, animals infected with very low doses of WHV (<10^3^ virions) develop another form of persistent asymptomatic infection, termed as primary occult infection (POI), that progresses in the absence of WHsAg, anti-WHc and anti-WHs, but WHV DNA is detectable in serum at similar levels as in SOI (Figure 1B) (29, 30). This infection is accompanied by essentially normal liver biochemistry and histology but HCC may develop (31). The human equivalence of this infection is seronegative OBI (28, 32). More details on the natural history and characteristics of SOI and POI are given in sections on pages 3 and 5, respectively, while features of coinciding immunological responses are summarized in section beginning on page 7.

## Persistence of Pathogenic WHV After Resolution of Symptomatic Infection: Secondary Occult Infection (SOI)

**Figure 2:**
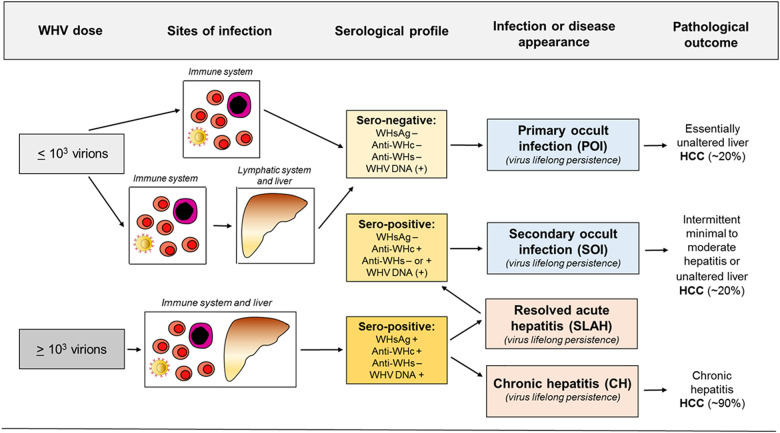
Abbreviated concept of the development of occult and overt (symptomatic) WHV infections, sites of replicating virus, and serological appearance and infection outcomes in relation to a dose of invading virus. For details see text.

HBV persistence in the absence of serum HBsAg, biochemical indicators of liver injury and clinical symptoms was rarely reported prior to introduction of HBV DNA detection by nucleic acid amplification tests (NAT) (33–36). Application of an increasingly sensitive and controlled NAT made identification of serologically silent HBV infection (i.e., OBI) more frequent and reliable (27, 37–40). With time, OBI became defined as persistence of HBV DNA in the absence of serum HBsAg. It later became apparent that this asymptomatic infection may have severe clinical consequences due to reactivation of hepatitis following immunosuppression, radiation or administration of immunomodulatory and cytotoxic therapies (41–45). With accumulation of molecular and immunological evidence, including data from the woodchuck model (28–30, 32, 46, 47), it became apparent that there are two distinct forms of OBI (Figures 1, 2). Both are serum HBsAg-negative but one is accompanied by anti-HBc with or without detectable anti-HBs, and another in which the antibodies are absent (13, 32).

One of the earliest studies demonstrated HBV persistence in serum and peripheral blood mononuclear cells (PBMC) collected from seemingly healthy persons after spontaneous resolution of AH type B (27). In these individuals, HBV DNA remained detectable in both serum and PBMC in the presence of anti-HBs and anti-HBc antibodies for up to 70 months after clinical resolution of AH. HBV DNA was detected by nested polymerase chain reaction (PCR) with primers specific for different HBV genes. Specificity of HBV amplicons was verified via nucleic acid hybridization (NAH). The estimated sensitivity of the PCR-NAH assays was <10 vge/mL. It was also found that HBV DNA in plasma co-sedimented with HBsAg reactivity in sucrose gradients and that some particles reactive for HBV DNA displayed buoyant density of intact virions in cesium chloride gradients. It was postulated that traces of infectious HBV persist after termination of SLAH in these otherwise healthy individuals. The existence of asymptomatic HBV infection after resolving AH was confirmed in subsequent studies [e.g., Penna et al. (48), Rehermann et al. (37), Uemoto et al. (38), Yotsuyanagi et al. (49), Yuki et al. (40), Murakami et al. (50)].

The findings from the 1994 study (27) triggered investigations in the woodchuck model which have brought a number of important discoveries. These findings have solidified data on the existence, molecular and immunological characteristics, and pathogenic significance of occult hepadnaviral infection continuing after resolution of a symptomatic disease. Because this form of infection follows clinically and serologically apparent infection, it was designated as SOI (secondary occult infection) (5, 30) and later also referred to as seropositive-OBI since antibodies to viral antigens remained detectable (Figures 1A, 2) (28).

Studies in woodchucks revealed that silent replication of WHV persists for life after recovery from AH and that SOI can culminate in the development of HCC (Figure 2) (29). It was also uncovered that both the liver and the immune system are the sites of virus replication. Thus, after resolution of AH, the serum levels of WHV DNA oscillated from <10 to 100 vge/mL, with occasional rises up to 10^3^ vge/mL. However, there were periods when serum WHV DNA was negative, but virus genome could be detected in the liver biopsy and/or PBMC acquired at the time of serum collection. WHV DNA quantities in hepatic tissue, PBMC and lymphatic organs, such as spleen, lymph nodes and bone marrow, ranged between 0.02 to 200 vge/10^4^ liver cells and 0.005 to 0.5 vge/10^4^ lymphoid cells. WHsAg in serum remained negative during entire SOI, while anti-WHc were consistently detectable during lifespan (Figure 1A). Histological examination of serial liver biopsies revealed minimal-to-mild inflammation with periods of essentially normal liver morphology. However, HCC ultimately developed in about one fifth of animals (Figure 2). Importantly, WHV carried by animals with SOI induced typical AH in virus-naïve animals which advanced to CH and HCC in some woodchucks (29). These data unambiguously showed that resolution of AH does not reflect complete elimination of virus and did not prevent HCC development, even when anti-WHs antibodies arise. These were unexpected findings considering the prevailing opinion at that time.

The subsequent study verified that detection of anti-WHc alone, in the absence of conventionally detectable serum WHsAg, indicates existence of SOI (51). Notably, electron microscopic examination of pellets from ultracentrifuged anti-WHc-positive sera demonstrated singular spherical and short tubular particles of WHsAg. This indicated that small amounts of the antigen were produced although they were not detectable by otherwise highly sensitive, clinically compatible assay. The results were consistent with other investigations demonstrating that the WHV persisting in animals with the anti-WHc alone remained infectious (29). They also supported the notion that persistence of anti-core alone is a consequence of re-stimulation of the immune system by traces of nucleocapsid protein transcribed during subdued virus replication. Thus, the anti-core alone showed to be an excellent marker of enduring hepadnaviral replication accompanied by quantities of circulating virus DNA which may not be consistently detected due to their fluctuating levels.

## Traces of WHV Induce Primary Occult Infection (POI) That is Persistent, Primarily Lymphotropic and May Cause HCC in the Absence of Hepatitis

POI was originally discovered in offspring born to woodchuck mothers which resolved AH and established SOI, including those which developed anti-WHs (46). All of the offspring demonstrateated low levels of circulating WHV DNA and WHV DNA, covalently closed circular DNA (cccDNA) and mRNA in the lymphatic system, whereas hepatic tissue was infected only in some of them. Surprisingly, no markers of WHV infection, including serum WHsAg, anti-WHc or anti-WHs, could be detected after birth (Figure 1B). Nonetheless, particles reactive for WHV DNA with biophysical features of intact (enveloped) virions were found in offspring sera. Histological examination of serial liver biopsies revealed normal morphology. Further, sera and supernatants of cultured lymphoid cells from these animals induced typical serologically apparent AH in WHV-naive animals which verified pathogenic fitness of the persisting virus. Remarkably, the offspring were not protected from challenge with a large, liver pathogenic dose of WHV [see (30)]. This implied that this low level infection observed in offspring did not generate immunity against reinfection with larger doses of the same virus (5, 46). The study was the first to uncover that hepadnaviral infection can be restricted to the immune system arguing that hepadnavirus at low quantities is preferentially lymphotropic (Figure 2).

The subsequent study was designed to determine if POI can be established in adult immunocompetent woodchucks and to find WHV dose required to induce POI. For this purpose, adult healthy animals were intravenously (*i.v*.) injected with serial 10-fold dilutions of a well-defined WHV inoculum known to cause serologically evident infection and AH (30). The results of the study not only confirmed the existence of this form of occult infection, but also showed that the infection is triggered by WHV doses lower than or equal to 1 × 10^3^ virions and can be reproducibly established in woodchucks. The same study demonstrated that the same WHV inoculum that caused POI induced at WHV doses larger than 1 × 10^3^ virions classical WHsAg-positive AH that advanced to CH in some animals. Taken together, the data revealed that: (1) the amount of virus is the principal determinant of whether infection is serologically evident (overt) and symptomatic or serologically silent and asymptomatic (occult); (2) virus at quantities >1 × 10^3^ virions causes WHV hepatitis, and (3) the immune cells are primary targets and can be the only site of replication when virus invades at doses smaller than 1 × 10^3^ virions (Figures 1, 2).

**Figure 3:**
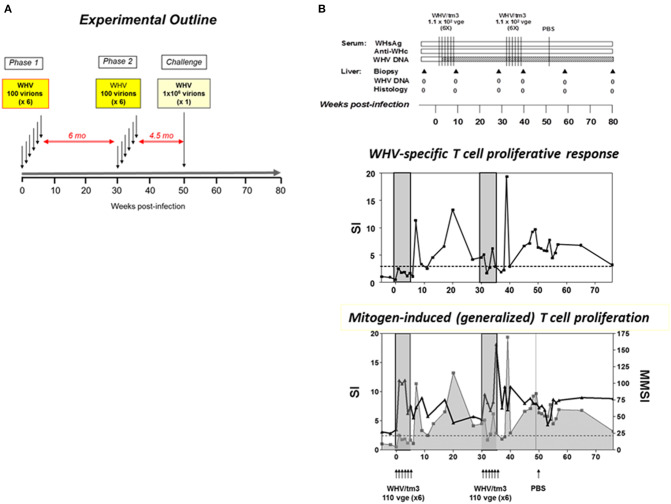
Outline of the study investigating virological, immunological and liver injury outcomes in woodchucks injected with multiple liver-nonpathogenic doses of WHV. **(A)** Four WHV-naïve, healthy animals were intravenously injected with 6 weekly doses of 110 WHV virions (Phase 1). Six months later they were challenged with another 6 weekly doses of 110 virions each of the same virus (Phase 2). The total quantity of the virus administered was 1,320 virions and cumulatively exceeded a liver pathogenic dose of 1 × 10^3^ virions. Three-and-half months later, the animals were re-challenged with either a liver pathogenic dose of 1.1 × 10^6^ virions of the same virus or injected with sterile PBS as controls (Challenge). **(B)** An example of the outcomes investigated in a woodchuck subjected to two rounds of injections with 6 weekly 110-viron doses of WHV and then injected with PBS. The data showed the lack of appearance of serologically detectable infection, including absence of anti-WHc antibodies which are very sensitive indicator of exposure to WHV, or hepatitis (upper panel). WHV-specific T cell response (middle panel) was preceded by ConA-induced, generalized T cell proliferation (lower panel). For details and abbreviations, see the legend to Figure 4. Figures adopted with modifications from Gujar et al. (52).

It was further examined whether repeated exposures to trace amounts of WHV normally establishing POI could result in serologically apparent infection and hepatitis. Adult animals were i.v. injected with twelve 110-virion doses over two rounds of 6 injections delivered weekly (52). At 14 weeks after the last injection, the animals were challenged with the same WHV inoculum at 1.1 × 10^6^ virions and followed for additional 7.5 months (Figure 3). The results showed that the multiple injections of liver non-pathogenic virus did not culminate in serum WHsAg and/or anti-WHc positive infection and hepatitis, even though the overall amount of administered WHV was greater than the threshold of 1 × 10^3^ virions known to trigger hepatitis. Not surprisingly, the animals remained susceptible to challenge with a liver pathogenic dose of WHV and responded by establishing AH (52). Repeated exposures to trace quantities of HBV may occur in different occupational and familial situations and in intravenous drug users. The results of this study suggest that such multiple exposure will unlikely cause serologically evident infection and hepatitis in individuals not protected by vaccination, but HBV DNA in the absence of HBsAg, anti-HBc and anti-HBs might be detected.

To recognize the life-long consequences of POI, young adult woodchucks were investigated in a prospective study in which POI was established by i.v. injection with 10 or 100 DNase digestion-protected virions (31). Initially, WHV infection was constrained to cells of the lymphatic (immune) system in all animals. Serum WHV DNA loads were between <100–200 vge/mL during lifespan and <1 × 10^3^ vge/μg of cellular DNA in PBMC. After 2.5 to 3.3 years post-infection (p.i.), WHV infection spread to liver and low levels of WHV DNA, cccDNA and/or virus mRNA became detectable despite that hepatic histology remained normal. Notably, temporal increases in serum WHV load above 1 × 10^3^ vge/mL preceded the involvement of the liver in infection. Among other original findings was that 20% of the animals with POI finally developed HCC. This implied that the virus persisting in POI maintained its pro-oncogenic potency and led to HCC in spite of the lack of hepatitis or other protracted liver injury. This was consistent with the finding that POI was accompanied by WHV DNA integration to hepatic, immune organs and PBMC genomes (31). This study pointed to the pathogenic significance of POI and implied that the development of HCC of unknown etiology in humans should consider this clinically and serologically mute infection as a causative factor (54, 55).

## Systemic and Intrahepatic Immune Responses in Pre-Acute Infection and Acute WHV hepatitis

Our step-by-step understanding of the events coinciding with the initiation of HBV infection and hepatitis is hindered by the lack of recognition of processes occurring in the liver and the immune system immediately following invasion with virus and during the early pre-acute and acute periods of infection. Identification of patients with these essentially asymptomatic stages of infection is excitingly difficult and collection of liver samples practically impossible because of the absence of clinically sound indications. However, these early events are likely of primary importance for identification of immunological and other factors determining the development of symptomatic or occult infection, recovery from AH or its progression to CH, and initiation of pro-oncogenic processes which may culminate in HCC (56–58). The woodchuck model is particularly well-suited for this type of studies.

The hepatic kinetics of WHV replication and transcription of the genes encoding cytokines, immune cell markers and cytotoxic effector molecules, as well as the profiles of WHV-specific and generalized (mitogen-induced) T cell responses have been delineated in experimentally infected woodchucks soon after virus administration and in the very early stages of infection. Serial liver biopsies acquired from 1 h p.i. up to 36 months p.i., when AH resolved and SOI was established, were analyzed by quantitative real-time PCR assays preceded by the reverse transcription step (RT-qPCR) (17). The results uncovered that WHV replication became detectable in the liver in an hour after i.v. injection with virus. This was in contrast to the previous studies in woodchucks and HBV-infected chimpanzees indicating that virus replication remains undetectable in the liver until 3–4 week p.i. (15, 59, 60). However, hepatic replication of these viruses was never before evaluated in the first hour p.i. by highly sensitive PCR-NAH assays. Briefly, it was found that in 3 to 6 h p.i., hepatic transcription of interleukin (IL)-12, which is a key cytokine produced by antigen presenting cells (APC) (61), was significantly (~20-fold) augmented together with an increase (12.5-fold) in IL-8 expression, a cytokine mediating chemotaxis of phagocytic cells. This also coincided by increased expression of CD1d, a molecule facilitating antigen presentation by APC to natural killer (NK) T cells (62), and CD40 ligand (CD40L), which is implicated in APC activation via CD40-CD40L interaction. In 48 to 72 h, NK and NK T cells became active as implied by significant increases in hepatic expression of CD3, IFN-γ, OAS (2′,5′-oligoadenylate synthetase), CD1d and CD40L, and in NKp46 and perforin expression. Most interestingly, WHV replication was significantly inhibited at the same time in the liver, implying that this early innate intrahepatic response was at least partially effective in inhibiting WHV replication (17) Nonetheless, hepatic CD4+ and CD8+ T cells became robustly activated much later at 4 to 5 weeks p.i. when hepatitis became evident. Overall, the findings showed that liver replication of WHV is initiated and the innate response activated very soon after infection with a liver pathogenic dose of WHV (30). Nevertheless, this transient response was insufficient to right away activate T cells similarly as in other viral infections. In a somewhat similar study in chimpanzees in which liver biopsies were collected from 1 week post-HBV injection, hepatic tissue did not express of genes coinciding with innate immune response when evaluated by microarray (60). The conclusion was that HBV is a stealth virus unable to induce innate immunity in the infected host. However, activation of the innate response may have occurred earlier and already subsided at 1 week, as it was apparent in the WHV infection model. Subsequent studies showed HBV ability to trigger different branches of the innate response, although it remains not well-explained what their effector functions are in hosts naturally susceptible to HBV infection [reviewed in Thomas and Baumert (63)].

**Figure 4:**
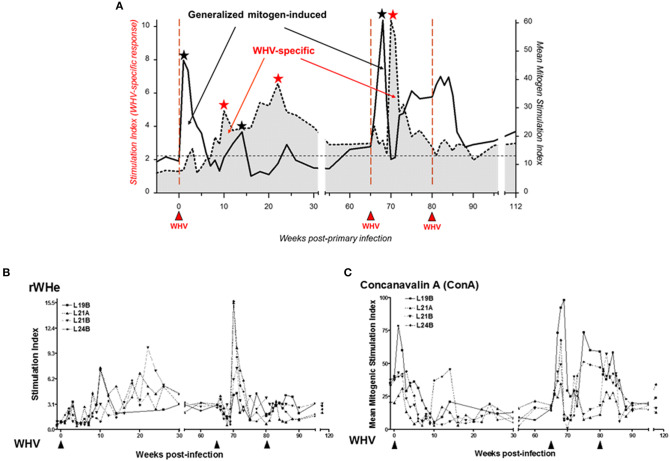
Distinct kinetics of WHV-specific and generalized T cell proliferative response following infection with a liver pathogenic dose of WHV and subsequent challenge with the same ioclum. **(A)** Schematic depiction of the cumulative data on discordance between virus-specific and generalized, mitogen-induced T cell proliferative responses. The profiles were compiled based on the data obtained from 4 animals with self-limited acute hepatitis after infection with 1.9 × 10^11^ DNase digestion-protected WHV virions using a flow cytometry CFSE assay to assess T cell proliferation in response to five different recombinant or native WHV proteins and WHc_97−110_ peptide (stimulation index values, SI) and in response to five 2-fold dilutions of mitogen concanavalin A (ConA) ranging from 1.25 to 20 μg/mL measured by a [^3^H]-adenine incorporation assay (mean mitogen stimulation index values, MMSI). The mean of the highest SI given by any WHV antigen or that of MMSI in response to any ConA concentration from each of four animals were used to construct the profiles. Solid red upward arrowheads indicate injections with WHV. Black and red stars mark peaks of mitogen-induced and WHV-specific T cell responses, respectively. The data demonstrate that WHV first triggers polyclonal generalized activation of T cells and then WHV-specific T cell response. **(B)** An example of the WHV-specific T cell response to recombinant WHV e protein (rWHe) in the same 4 animals shown in panel A. **(C)** An example of the mitogen-induced T cell proliferation after infection and challenge with WHV in response to stimulation with ConA. Figures adopted with modifications from Gujar et al. (53).

The common characteristic of HBV and WHV infections is the postponement of virus-specific T cell response and the reason behind this was unknown. This is in contrast to infections with other viruses where specific T cells usually appear in about 10 days to 2 weeks p.i. To investigate this issue, woodchucks were inoculated with 1.9 × 10^11^ WHV virions and challenged with the same virus dose several weeks later (53). As anticipated, the WHV-specific T cell response appeared 5 to 7 weeks p.i., remained elevated during AH, and then subsided but remained measurable during SOI. However, soon after administration of WHV, i.e., in the first 7 days p.i., T cells demonstrated significantly augmented proliferation in response to mitogenic stimuli, i.e., concanavalin A (ConA), pokeweed mitogen (PWM), and phytohemagglutinin (PHA) (Figure 4). Thus, this strong antigen-nonspecific, generalized T cell response occurred before the appearance of WHV-specific T cell reactivity, which was previously unknown consequence of hepadnaviral infection. Analysis of cytokine transcription in weekly PBMC samples confirmed very early activation of T lymphocytes and impairment in transcription of tumor necrosis factor-alpha (TNF-α) and IFN-γ up to week 6 p.i., thus until AH and WHV-specific T cell response became apparent. The pre-acute phase of WHV infection was also associated with reduced transcription of IFN-α, IL-2, and IL-12, and increased expression of IL-10. This cytokine expression profile was compatible with the profiles observed in the early infections with other viruses and was found to be indicative of defective activation of APC and presentation of viral epitopes to T cells (64, 65).

Interestingly, re-exposure of woodchucks which resolved AH to the same WHV again immediately triggered strong, generalized proliferative T cell response which was followed by the delayed virus-specific T cell reactivity (53). It is commonly acknowledged that re-exposure of immunocompetent hosts to the same biological agent swiftly mounts secondary adaptive response detectable within a week or two after re-challenge [e.g., Flynn et al. (66), Doherty et al. (67), Rocha and Tanchot (68)]. In this context, the results obtained suggested that temporal, generalized, polyclonal activation of T cells delayed adaptive T cell response not only after primary but also after secondary exposure to WHV. This was the first of this kind finding that was not yet examined in HBV infection.

It was noticed that the generalized proliferative capacity of T cells varied depending on the phase of acute WHV infection (53, 69) and was significantly heightened promptly after infection and then dramatically declined before WHV-specific T cell response appeared (Figure 4). Hence, it became of interest to examine what the mechanism of this rapid T cell decline was. For this purpose, a flow cytometric CFSE [5-(and-6)-carboxyfluorescein diacetate succinimidyl ester]-based assay coupled with staining for annexin V-7 in the presence of actinomycin-D (7-AAD) was adopted to simultaneously measure lymphocyte proliferation and apoptotic lymphocyte death (53, 69, 70). The evaluation of lymphocyte behavior after WHV infection leading to SLAH showed that the rapid contraction of the generalized T cell response was associated with the strongly augmented susceptibility of T cells to activation-induced apoptotic death. This indicated that T cells became compromised in the early phase of acute WHV infection and this may directly contribute to the postponement of WHV-specific T cell response.

In summary, both primary and secondary exposures to liver-pathogenic doses of WHV caused generalized activation of T lymphocytes accompanied by the cytokine expression profile suggesting defective activation of APC and priming of virus-specific T cells. These processes occurred in the initial phase of infection and were likely responsible for postponement of WHV-specific T cell response. Consequently, this implied that the hindered initial recognition and elimination of virus permitted its replication and spreading in the very early, asymptomatic phase of infection. Similar findings were reported in the early infection with other viruses, including simian immunodeficiency virus (SIV) which infects T cells [e.g., Wallace et al. (65)]. As previously mentioned, WHV and HBV are lymphotropic viruses which infect cells of the immune system. For more details on WHV lymphotropism see section on page 9.

## WHV-Specific T Cell and Humoral Immune Responses in Chronic Symptomatic and Persistent Occult Infections

CH remains the main burden of HBV infection despite significant progress in treatment options. The woodchuck model meaningfully contributed to identification of features of virus-specific T cell responses during symptomatic chronic infection and recognition of ways to modulate them for therapeutic purposes. Due to a very weak or absent activity of these responses, their augmentation either alone or in combination with antiviral agents may offer a prospect of more effective therapy for CH type B.

Identification of immunodominant T cell epitopes within WHV core and envelope proteins, and development of the first generation of assays measuring helper and CTL responses initiated recognition of the anti-WHV cellular immunity in woodchucks (14, 71, 72). These first tests included 2[^3^H]-adenine incorporation T cell proliferation assay and flow cytometric CD107a degranulation test to assess WHV-specific cytotoxic T cells (CTL) response (14, 15, 72). The data showed a close compatibility between woodchucks and humans in adaptive T cells responses both during resolution of AH and CH. Thus, recovery from AH coincided with strong T cell proliferative and CTL reactivity directed to multiple WHV antigenic epitopes which coincided with rapid decline in circulating virus. In contrast, weak or undetectable T cell responses were identified in CH in the presence of usually high loads of circulating WHV (14, 71, 72). With time more sensitive or applying different approaches assays were developed, including a flow cytometric CFSE-based assay capable of evaluation of relative strength and kinetics of WHV-specific T cell proliferation (52, 53, 70) and flow cytometric assays quantifying WHV-specific CTLs based on defining CD3-positive, CD4-negative (73) and CD4-negative, IFN-γ positive T cell subsets (Mulrooney-Cousin and Michalak, unpublished). The results from these assays remained in good agreement with the findings previously reported.

The weakened or absent virus-specific T cell response in chronic infections was found to be a consequence of T cell exhaustion in which programmed death 1(PD-1)/PD-ligand 1 (PD-L1) interaction plays a dominant role [e.g., Barber et al. (74), Maier et al. (75), Velu et al. (76)]. The restoration of this interaction *in vitro* brought promising results, however experiments with PD-1 blocking anti-PD-L1 antibodies alone *in vivo* were not as much successful (77, 78). Chronically infected woodchucks, like HBV-infected humans, can have elevated liver PD-L1expression and increased display of PD-1 on CD8+ cytotoxic T cells. Woodchuck PD-1 and PD-L1 and PD-L2 were cloned and characterized, and antibodies against PD-L1 produced (18, 73). Function of WHV-specific CTLs was significantly enhanced *in vivo* in some woodchucks with CH when anti-PD-L1 antibodies were given together with entacavir (ETV), a clinically used anti-HBV nucleoside analog, and DNA vaccination with plasmids expressing WHc and WHs antigens (19). In more recent study, the effect of anti-PD-L1 in combination with ETV was only seen in a minority of chronically infected animals (73). Nonetheless, this approach may represent valuable therapeutic strategy for CH type B after further improvements in consistency and durability of the T cell response.

SOI continuing after recovery from an episode of AH is associated with low levels of T cell response toward WHV antigenic epitopes which is intermittently detectable throughout lifetime (Figure 4). This profile of T cell reactivity during SOI closely resembles the profiles of proliferative and CTL responses against HBV in patients who resolved AH type B (37, 48) who, like woodchucks, continue to carry after SLAH traces of replicating virus for years. It is now acknowledged that the residual transcription of small amounts of viral proteins provides continuous antigenic stimulation that maintains an active antiviral immune response during occult infection. This response sustains persisting virus at levels which may no be longer liver pathogenic; however, this control may fail and reactivation of hepatitis may occur (32, 45).

The features of WHV-specific T cell response were also investigated in POI and after challenge of woodchucks with POI with liver pathogenic or non-pathogenic doses of WHV (79). Similarly as AH, POI was associated with the delayed appearance of WHV-specific T cell proliferative response against multiple virus epitopes (53). This T cell reactivity persisted intermittently at low levels as it was seen in the course of SOI. Like in WHV AH, immediately after inoculation with WHV establishing POI, lymphocytes displayed an augmented capacity to proliferate in response to mitogenic stimuli prior to arise of virus-specific response (79). Interestingly, the profiles of both virus-specific and generalized T cell proliferative responses were again very similar to those observed after infection with liver pathogenic doses (Figures 3, 4). These results well-supported the view that WHV-specific T cell reactivity is an extremely sensitive indicator of exposure to hepadnavirus, even to amounts as low as 10 virions (31). However, there were two major differences between POI and SOI considering immune response. In contrast to SOI, POI was not accompanied by anti-viral antibodies, including anti-WHc which as anti-HBc normally accompany WHV or HBV infection. Another distinctive feature was that POI did not induce protective immune response against WHV, while infection with liver-pathogenic doses leading to SOI invariably did (52, 53, 79). This confirmed a central role of humoral anti-viral immunity in protection against re-exposure to hepadnavirus. Overall, the discrepancy between virus-specific cellular and humoral responses to infection with a low dose of WHV was consistent with the data from other asymptomatic infections, including those with hepatitis C virus, human immunodeficiency virus type 1 or SIV (80–84). In these infections, T cell responses occured in the absence of serological or pathological signs of infection. Comparable findings were reported for humans exposed to HBV (85).

## The Immune System as Site of Replication and Lifelong Persistence of WHV

It became evident during studies of the woodchuck model that the immune system is the site of productive WHV replication as well as reservoir of persisting virus independent of whether infection is symptomatic or occult (Figure 2). Infectivity of WHV derived from lymphoid cells of infected animals and from *in vitro* infected immune cells have been comprehensively documented and reviewed (5, 9, 29–31, 46, 47, 86–93)].

Among others, the method of WHV DNA identification within intact lymphoid cells by employing *in situ* PCR combined with flow cytometric quantification of cells containing WHV amplicons was established to enumerate infected cells (91). The data showed that 3.4 to 20.4% (mean 9.6%) of the circulating lymphoid cells carried WHV DNA in animals with AH and CH, while cells from SOI or POI were positive at lower numbers ranging from 1.1 to 14.6% (mean 4.4%) (91). There was no meaningful difference in numbers of lymphoid cells carrying WHV genome between SOI and POI, which was consistent with comparable loads of WHV DNA in PBMC as measured by PCR-NAH (29, 30, 46, 94, 95).

To investigate whether WHV ability to infect immune cells is a feature of wild-type virus or a result of a particular virus variant predisposed to infect these cells, WHV with a homogenous sequence in both the liver and the immune system was subjected to serial passage in woodchuck hepatocytes and splenocytes (92). It was hypothesized that such repeated passage should enrich a lymphotropic variant if it exists. This repeated passage did not lead to the appearance of cell type-specific WHV variants, has not changed virus cell tropism or its infectivity when administered to WHV-naïve woodchucks. The resulting infection profiles in the animals infected dependent upon virus dose but not on virus cellular origin, and the virus retained its initial DNA sequence.

Although hepatocytes and lymphoid cells are targets of HBV and WHV (96–109), there is very limited knowledge regarding the receptor/s determining these viruses lymphotropism. However, it was demonstrated that there is a protease-activated cell binding site in the large preS1 protein of WHV envelope that facilitates *in vitro* binding to woodchuck hepatocytes and lymphoid cells (110, 111). The core epitope of this site was mapped to amino acid residues 10–13 at the N-terminal sequence of the preS1 protein. Peptides comprising this site sequence interacted with woodchuck hepatocytes and lymphoid cells with characteristics of a specific ligand-receptor interaction. Interestingly, their ability to bind lymphoid cells was about 1000-fold greater than that for hepatocytes. This cell recognition site was found to be protected within the WHV preS1 protein tertiary structure and its activity was not identifiable until the protein was subjected to limited digestion with proteases. The antibodies directed against the site appeared as the first immunological indicator of infection in animals experimentally infected with WHV, indicating that proteolytic cleavage of this protein also occurred *in vivo* (111). In addition, the involvement of a 330-kD proteoglycan, as well as heparan sulfate and polymannose in the binding of WHV envelope to hepatocyte and splenocyte plasma membranes was described (112).

## Interactions Between WHV Proteins and Hepatocyte Surface Plasma Membrane and Their Significance

**Figure 5:**
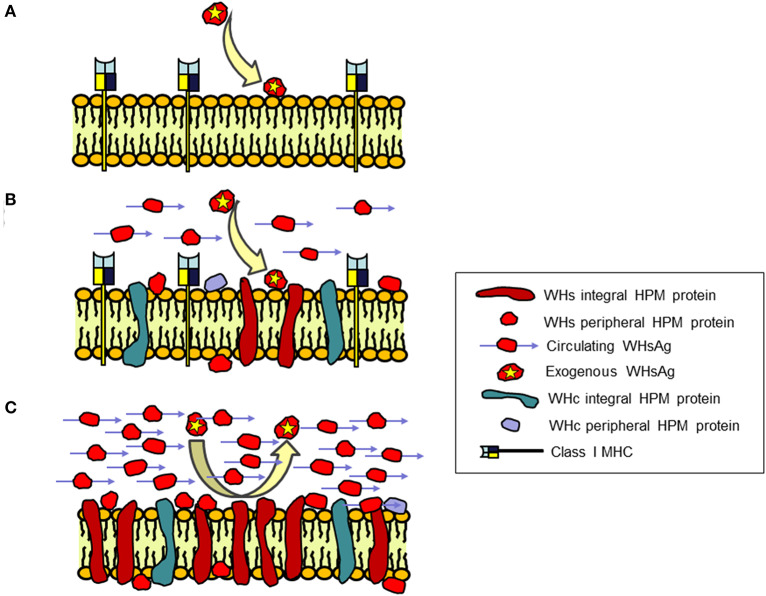
Schematic presentation of the concept of an immune protective barrier at the surface of hepatocytes in chronic WHV infection created by virus envelope (WHs) proteins associated with hepatocyte plasma membranes (HPM) that coincides with depletion of class I MHC on the membranes. HPM from **(A)** Healthy, WHV-negative woodchucks; **(B)** WHV-infected animals with acute hepatitis (AH), and **(C)** WHV-infected animals with chronic hepatitis (CH). CH in contrast to AH is characterized by irreversible incorporation of large (saturating) quantities of WHs proteins into HPM lipid bilayer and presence of WHs reactivity loosely associated with HPM structure behaving as peripheral membrane protein. In addition, CH occurs in the context of large quantities of circulating virus envelope proteins carrying WHs antigenic specificity (WHsAg) and inability of HPM to bind exogenous purified WHsAg in contrast to HPM from healthy woodchucks and animals with AH. WHV core (WHc) proteins are also incorporated as integral and joined with HPM as peripheral membrane proteins, however there are no differences between AH and CH in their HPM quantities and susceptibility to removal by agents dissociating HPM. In addition, HPM from CH are voided of class I MHC presence in contrast to HPM from acutely infected and healthy animals. Together, the concept assumes that WHs proteins incorporate at saturating quantities into hepatocyte surface membranes in combination with large amounts of circulating WHsAg and absence of class I MHC presentation at the membranes form an immune protective barrier that shelters infected hepatocytes from immune elimination. For details see Michalak and Churchill (115), Michalak et al. (116, 117), Michalak and Lin (118), Michalak et al. (119), and Wang and Michalak (120).

Hepatitis caused by hepadnaviral infection is in principle a result of the immune response directed against viral peptides carrying epitopes which are presented at the hepatocyte surface in the context of major histocompatibility complexes (MHC). These epitopes are recognized by the epitope-specific CD4+ and CD8+ T lymphocytes which perform supportive (helper) and cytotoxic functions directed against infected cells, respectively (113, 114). However, viral proteins are also directly incorporated into hepatocyte plasma (surface) membranes (HPM) as integral proteins or loosely linked with the HPM structure as peripheral plasma membrane proteins (Figure 5). These HPM-associated viral proteins can themselves serve as targets of cytopathic reactions and potentially modify the extent of immune-mediated liver damage.

In the HBV infection known as asymptomatic, healthy chronic carriage of HBsAg and in the equivalent infection in woodchucks, normal or nearly normal liver histology and unaltered hepatic biochemical functions coincide with the vast quantities of circulating HBsAg or WHsAg, respectively (121, 122). This is accompanied by abundant deposits of virus envelope material in the cytoplasm and in the proximity of the hepatocyte surface in the majority, if not in all, hepatocytes. This naturally occurring situation seemingly perfectly exemplify the state of immune tolerance in the presence of excess of foreign immunogen. The above observations triggered a question whether there is a relationship between the status of hepadnavirus protein association with HPM and the form of inflammatory liver injury.

In a series of studies, HPM purified from woodchucks with different immunomorphological forms of WHV hepatitis and HCC were investigated for the nature of WHV proteins' interaction with the HPM lipid bilayer, the amounts and the molecular profiles of the associated WHV proteins, and the ability of exogenous WHsAg to interact with HPM (115–118). Regarding WHV nucleocapsid (also called core or WHc) proteins, their levels and molecular species displayed by infected HPM were not related to form, severity or duration of hepatitis (Figure 5) (118). Notably, two WHc proteins with molecular weights of 22 and 43 kD were identified in HPM from both AH and CH. Quantitation of the WHc content in HPM from AH (mean 1.62 ± 0.96 ng/μg HPM protein) and CH (mean 1.39 ± 0.39 ng/μg HPM protein) were not statistically different (118). Conversely, quantities of the WHs proteins were meaningfully greater in HPM from CH (mean 16.36 ± 2.05 ng/μg HPM protein) than those from AH (mean 2.60 ± 1.15 ng/μg HPM protein) (Figure 5) (118). Molecular profiles determined by Western blotting showed that the WHV preS1, preS2, and S polypeptides, with or without equivalents in the purified serum WHsAg and WHV virions, were detected in all infected HPM. Detection of the WHs polypeptides other than those comprising circulating particles implied that the proteins unique for intracellular processing and not utilized in assembly of viral particles were inserted into HPM. Further, staining with antibodies against WHsAg which recognized all envelope proteins and with monoclonal antibody against WHV preS2 peptide demonstrated that the preS2 protein was dominant in all infected HPM (118).

Examination of HPM from animals with AH or CH demonstrated that most of WHc proteins resisted extraction with 6 M urea that removes peripheral membrane proteins loosely associated with HPM, indicating their strong bond with the HPM lipid bilayer. Treatment of HPM with Triton X-114 (1%), which dissociates membranes into the hydrophilic and the hydrophobic protein fractions, released the majority of WHc polypeptides confirming their nature as integral proteins. There was no difference in the behavior of the HPM-associated WHc proteins from animals with diverse severity of AH or CH (118).

The majority (~85%) of the proteins carrying WHs specificity was very tightly incorporated into HPM and resisted treatments with strong plasma membrane perturbants (115, 118). Extraction with agents solubilizing the lipid bilayer, such as Triton X-114, Triton X-100 (1%) or deoxycholic acid (50 mM), ascertained their behavior as the integral HPM protein, indicating that their insertion was irreversible (115). This suggested that perhaps only lysis or apoptotic death of infected cells could eliminate these proteins. Furthermore, HPM from animals with CH, but not from healthy woodchucks or these with AH, were unable to bind purified exogenous WHsAg (Figure 5) (118). Similar incapability characterized HPM from chronic WHsAg carriers (123), implying that the HPM potential to interact with WHV envelope was exhausted. This was likely due to a large amount of WHs proteins already incorporated into and associated with HPM and occupation of hepatocyte virus receptors by virus envelope material. However, such exhaustion could also be due to formation of WHsAg-anti-WHs immune complexes. Circulating anti-WHs or anti-HBs antibodies are rarely detectable in CH, however such possibility exists and a contribution of HBsAg-anti-HBs immune complexes to hepatocyte injury in CH was postulated (124). From this perspective, HPM eluates from woodchucks with AH contained immunoglobulins displaying anti-WHc and anti-WHV e antigen (anti-WHe) reactivity, although anti-WHs antibodies were not detected (117).

Taken together, the irreversible incorporation of WHV envelope but not core proteins into HPM lipid bilayer was a specific characteristic of CH (Figure 5). The accumulation of WHs proteins in HPM was not related to CH duration, severity of inflammation or progression to HCC. This raised a concept that such a state could be linked with the development and protraction of liver disease perhaps by creating an immune protective barrier at the hepatocyte surface that shelters virally infected cells from immune elimination. Notably, the increased display of WHV envelope proteins, particularly pre-S2, was found later to coincide with an inhibition of class I MHC presentation on hepatocytes in CH (119, 120). This occurrence should impact the efficiency of immune elimination of infected hepatocytes by virus-specific CTL (also see section on page 12). Common immunovirological properties of CH and the host immune responses in WHV and HBV infections would suggest that comparable events may happen in CH type B. However, similar studies are not feasible in the HBV-infected individuals mainly due to the lack of liver tissue uncompromised by therapy, comorbidities and/or the-end-of-life events.

In addition to the expected immunomodulatory effect exerted by HPM-associated virus envelope proteins, viral proteins displayed at the hepatocyte surface can be targets of immunopathogenic reactions. As mentioned, the eluates of HPM from animals with AH demonstrated anti-WHc and anti-WHe but not anti-WHs reactivity, although all three antigenic specificities were detectable in HPM (117). Also, while anti-WHc was readily identifiable, anti-WHe could be detected only in eluates from HPM of animals recovering from AH (117). These findings indicated that hepadnaviral proteins associated with HPM can be recognized by specific antibodies and became targets of hepatocytotoxic reactions. This concept was consistent with demonstration that heterologous anti-HBc and anti-HBs, as well as antibodies to hepatocyte asialoglycoprotein receptor (ASGPR) were cytopathic in the presence of active complement when hepatocytes from treatment-naïve patients with CH type B were used as targets (125). The data also indicated that anti-HBc-directed complement-mediated cytotoxicity was augmented against hepatocytes from individuals with chronic active hepatitis over that against hepatocytes from chronic mild hepatitis or inactive cirrhosis due to HBV infection. Overall, the data suggested that both HBV proteins and ASGPR (also see section on page 12), are recognized by circulating antibodies and that antibody-mediated cytotoxicity may contribute to hepatocyte damage in HBV infection.

## Inhibition of Class I MHC Presentation on Hepatocytes in Chronic WHV Hepatitis

Generation of an antibody against a non-polymorphic epitope of woodchuck class I MHC heavy chain allowed identification of the MHC localization by flow cytometry and immunoblotting methods in woodchucks (126). Presentation of class I MHC was found on normal woodchuck hepatocytes. This was in contrast to the previous assumption based on immunohistochemical staining that normal hepatocytes do not display this complex. Further study of hepatocytes and HPM isolated from animals with WHV hepatitis revealed that AH, but not CH coincided with significantly augmented display of the class I MHC (119). This was associated with the enhanced expression of genes encoding for class I MHC heavy chain, ß_2_-microglobulin, transporters associated with antigen processing (TAP1 and TAP2), and IFN-γ. However, despite the similarly increased transcription of these genes in hepatocytes from AH and CH, the class I MHC display was suppressed only on hepatocytes from CH. Furthermore, the level of inhibition was not associated with the histological degree of hepatocellular damage, the severity of inflammation, the hepatic level of IFN-γ expression, and the liver load of WHV. This together implied a profound posttranscriptional inhibition in the class I MHC presentation on hepatocytes in CH and that this represents a common hallmark of CH (119). Interestingly, the class I MHC was also inhibited on splenocytes of chronically infected woodchucks which are also known to support WHV replication (29, 86, 92). Since, the class I MHC is principal to presentation of viral peptides to cytotoxic CD8+ T cells, this finding was of an utmost significance to the understanding one of the mechanisms responsible for development and endurance of CH in hepadnaviral infection. Because a similar study has not yet been accomplished in either human or any other animal model of hepadnaviral infection, it is appropriate to emphasize these results.

Subsequent investigations asked which of the WHV proteins could be responsible for the impairment of class I MHC presentation on hepatocytes. To study this issue, WCM-260 hepatocytes isolated from a healthy woodchuck (127, 128) were transfected with the genes encoding individual WHV proteins as well as with the complete WHV genome (120). It was found that the class I MHC display was significantly impaired after transfection with the entire WHV DNA or with genes coding WHV envelope preS2 middle or preS1 large protein, which also contains the preS2 protein. In opposite, transfection of hepatocytes with the WHV X gene alone augmented the class I MHC display, while hepatocytes expressing after transfection WHV major S protein or WHV core alone did not alter the class I presentation on WCM-260 hepatocytes. Interestingly but not surprisingly, hepatocytes treated with woodchuck recombinant IFN-γ reestablished the class I presentation suppressed by the transcription mentioned above (120). Overall, these data implied that the impaired display of class I MHC on hepatocytes transcribing WHV is a result of posttranscriptional inhibition due to an interference from the virus preS2 protein and that this impairment can be fully reversed by treatment with IFN-γ. In this regard, exposure of primary hepatocytes from WHV-infected woodchucks to woodchuck IFN-γ was also found to upregulate class I MHC transcription (129).

## WHV-Induced Asialoglycoprotein Receptor Autoimmunity and Its Effect on the Outcome of Hepadnaviral Hepatitis

The induction of humoral and cellular autoimmune reactions by viruses is well-documented, while the contribution of virus-induced autoimmunity to the development and maintenance of chronic liver diseases, including viral hepatitis, although postulated for a long time, remained sparsely recognized. Circulating autoantibodies to non-organ and liver-specific antigens frequently accompany CH type B (130, 131). Regarding liver-specific autoantibody responses, autoantibodies to hepatic ASGPR (anti-ASGPR) have been encountered in up to 73% of patients with CH type B (132, 133), but rarely in hepatitis C (<15%) (134). For comparison, approximately 80% of patients with chronic autoimmune hepatitis may carry anti-ASGPR (132, 135). The target for anti-ASGPR is a HPM-associated receptor, a hepatic C-type lectin, which facilitates removal through endocytosis and lysosomal degradation of desialylated glycoproteins carrying galactose-terminal oligosaccharides (136, 137). The structure and biological properties of ASGPR have been well-characterized for human and other species (136–138), including woodchuck (139). The woodchuck ASGPR (WcASGPR) is a hetero-oligomeric complex composed, similarly as human ASGPR, by two polypeptides with molecular masses of 47 and 40 kD (139). Since the expression of ASGPR is essentially restricted to hepatocytes and anti-ASGPR antibodies coincide at a high frequency with HBV infection, it was postulated that HBV may trigger anti-ASGPR which, in turn, could be implicated in liver damage (140). In this context, we have embarked to recognize whether hepadnaviral infection induces ASGPR autoreactivity and what consequences of this response are regarding hepatocyte function and liver pathogenicity in experimental CH. The woodchuck model is certainly well-positioned to answer such questions since a relationship between virus infection and development of autoimmunity can be investigated from the time of virus entry and easily related to the status of autoimmune response before infection. From this perspective, the woodchuck model has been previously used to investigate the impact of hepadnavirus on the induction of non-organ specific autoantibodies (141). It was uncovered that WHV infection undeniably induces a non-organ specific autoimmune response that appears during the incubation period before detection of serum WHsAg and clinical appearance of hepatitis. The presence of smooth muscle autoantibodies (SMA) was most pronounced among the autoantibodies tested, however their kinetics and levels did not differentiate weather AH resolves or progresses to CH.

The studies on induction of anti-ASGPR in the course of WHV infection and on potential pathogenic roles of this autoimmune response revealed that: (1) Inoculation with WHV triggered anti-ASGPR autoantibodies in healthy woodchucks at a frequency close to 90% (142); (2) Existence of anti-ASGPR reactivity prior to WHV infection was associated with development of CH at significantly greater rate (55.5%) than in woodchucks non-reactive for anti-ASGPR before infection (15.6%) (142); (3) WHV-induced anti-ASGPR antibodies inhibited recognition of asialoglycoprotein by woodchuck hepatocytes and human HepG2 cells suggesting that the induced anti-ASGPR might impair hepatocyte clearance of desialylated proteins (127), and (4) Anti-ASGPR antibodies triggered by WHV were able to induce complement-mediated hepatocytotoxicity implying that they may contribute to the pathogenesis, impact severity, and hinder recovery from liver damage in hepadnaviral hepatitis (127). In another related study, healthy woodchucks which were first immunized with a WcASGPR-closely compatible rabbit ASGPR (RbASGPR) and then infected with WHV advanced at a higher frequency to CH, while animals with ongoing CH challenged with RbASGPR demonstrated exacerbated histological degree of liver injury when compared to the unchallenged animals with CH (143). In general, the data pointed out that immune response directed to hepatocyte ASGPR has potential to modify the severity and the outcome of hepadnaviral hepatitis and likely liver status in other diseases accompany by this autoreactivity.

## Hepatocytes as Cytotoxic Effectors Capable of Cell elimination by Both Perforin and CD95 (FAS) Ligand-Dependent Pathways

**Figure 6:**
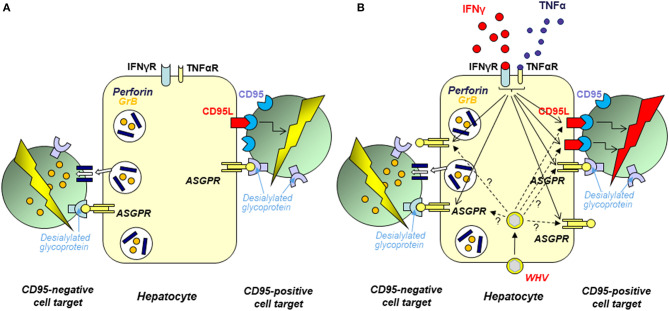
Schematic presentation of the mechanisms and factors affecting elimination of cells brought to contact with hepatocyte surface. In opposite to the previous opinion, hepatocytes intrinsically transcribe perforin, as well as CD95 and CD95 ligand (CD95L) and can utilize both perforin-granzyme B (GrB) and CD95L-CD95 pathways to kill other cells. The acquired experimental data indicate that hepatocytes can recognize other cells via interaction between desialylated glycoproteins on the target cell surface and asialoglycoprotein receptor (ASGPR) on hepatocyte plasma membrane. **(A)** Normal hepatocytes show ability to eliminate contacted cells by both CD95L- and perforin-dependent mechanisms. ASGPR involvement in this process is supported by demonstration that desialylation of surface glycoproteins on target cells enhanced their susceptibility to hepatocyte-mediated killing and that inhibition of hepatocyte ASGPR by asilofetuin, a ASGPR-specific ligand, or by silencing of ASGPR gene by specific siRNA significantly limited hepatocyte-facilitated cell killing. **(B)** The hepatocyte capacity to eliminate cells brought to contact with their surface is augmented after hepatocyte exposure to IFN-γ or TNF-α. This appears to be a consequence of the combined effects of enhanced expression of the cytotoxic effector molecules and augmented display of ASGPR on hepatocyte surface (indicated by arrows with continuous stamps). In chronic WHV hepatitis and resolved acute WHV infection, hepatocyte CD95L- and perforin-dependent cytotoxicity is augmented when compared to hepatocytes from livers of healthy woodchucks. This appears to be a consequence of liver inflammation caused by WHV which progression and resolution are associated with augmented production of many bioactive factors including IFN-γ and TNF-α. It also is possible that the augmented hepatocyte cytotoxicity in some situations could be due to a direct effect of virus (WHx protein) via upregulated expression of the cytotoxic effector molecules or ASGPR in hepatocytes (marked by arrows with dashed stamps). For details see Guy et al. (149–151), Guy et al. (152).

It is now acknowledged that the liver is an immunologically competent organ which plays important roles in maintenance of peripheral immune tolerance and surveillance over the gut originating pathogens. While the contribution of Kupffer cells and sinusoidal endothelial cells to the hepatic immune responses is relatively well-recognized (144–148), involvement of hepatocytes, which number reaches ~8 × 10^11^ and they comprise ~80% of the adult liver, remained unknown. We uncovered that hepatocytes can function as cytotoxic effectors and constitutively display ability to eliminate cells directly contacted with their surface *via* both perforin-granzyme B (GrB) and CD95 ligand (CD95L)-CD95 (formerly Fas ligand-Fas) death pathways (Figure 6) (149, 152). These were unexpected findings since the ability to eliminate cells *in vivo* was previously known only regarding immune cells, such as CTLs, which are endowed with the same cytotoxic mechanisms. Related data showed that hepatocyte cell killing can be differentially modified by cytokines. Thus, while IFN-γ and tumor necrosis factor alpha (TNF-α) upregulate hepatocyte expression and usage of CD95L, the ability of hepatocytes to eliminate cells via perforin-GrB was unaltered upon exposure to either cytokine (152) (Figure 6). Remarkably, it was also uncovered that hepatocyte cytotoxic potency depends upon interaction of terminally desialylated glycoproteins on target cells with ASGPR on the hepatocyte surface (150). While the most acknowledged role of ASGPR is the removal of desialylated glycoproteins (for more details see section on page 12), it has also been postulated that the receptor facilitates the trapping of activated lymphocytes in the liver. In this regard, it was reported that activated T cells carrying the B220 epitope, a CD45 molecule depleted of sialic acid, accumulate in the livers of CD95-deficient mice (153). Binding of activated T cells by hepatic ASGPR could facilitate the retention of the cells and their removal *via* an apoptotic mechanism involving the death signaling via CD95 (153, 154). Based on the data implying that hepatocytes can act as cytotoxic effectors, we hypothesized that they may play a role in removal of activated T cells (150). To test this possibility, we investigated whether hepatocyte surface ASGPR could be directly involved in recognition and removal of activated T cells. The results demonstrated that desialylation of glycoproteins on the surface of T cells augmented their hepatocyte-mediated apoptosis *in vitro*, while inhibition of hepatocyte ASGPR binding by a soluble ligand, such as asialofetuin, or silencing of the ASGPR gene by small interfering RNA (siRNA) significantly reduced hepatocyte-mediated cell killing. The study also revealed that hepatocytes can wipe out affinity-purified, mitogen-activated CD4+ T lymphocytes brought into contact with their surface (150).

It was further shown that both CH and resolved AH are associated with enhanced hepatocyte cytotoxicity that is dependent on an increased activity of both CD95L-CD95 and perforin-granzyme B mediated pathways (Figure 6) (151). This was supplemented by the study utilizing intact woodchuck WCM-260 hepatocytes and WCM-260 stably transfected with WHV DNA or singular WHV genes (120). We uncovered that hepatocytes treated with IFN-γ, but not those transfected with the complete WHV genome or individual WHV genes, excluding the WHV X gene, displayed augmented cytotoxicity facilitated by both CD95L and perforin. This implied that increased intrahepatic production of IFN-γ rather than virus replication itself led to increased hepatocyte-mediated cell death. In this context, an increased hepatic transcription of IFN-γ was reported in woodchucks during both CH and SOI continuing after resolution of AH, as well as in POI (16, 53). Interestingly, hepatocytes transfected with the WHV X gene alone displayed significantly higher levels of CD95L and perforin, and killed cell targets more efficiently (151). This suggested that WHV under certain circumstances may directly heighten hepatocyte cytotoxicity.

The studies summarized above for the first time revealed the cytotoxic phenotype of hepatocytes and showed that they are naturally equipped with the machinery facilitating death of other cells *via* perforin-granzyme B and CD95L-CD95-mediated pathways (Figure 6). The data also suggested that the recognition of target cells by hepatocytes involves ASGPR that recognizes desialylated glycoproteins on the surface of cells predestined for elimination. The appearance of these proteins is a natural outcome of the glycoproteins physiological usage, including activation of T lymphocytes (155–158). It was also shown that hepatocytes can directly eliminate activated T cells. This intriguing finding suggests that hepatocytes may actively contribute to local immune regulation and moderation of inflammation. Thus, hepatocytes might be much more than passive targets of immune reactions and may function as immunological effectors. Whether hepatocytes directly contribute to outcome of viral hepatitis *via* immunoregulation and/or contraction of inflammation remains to be established. Nonetheless, examination of the mechanisms underlying liver pathology should consider hepatocytes as potentially immunologically competent participants.

## Predictors of Spontaneous Resolution of Acute Infection and its Progression to Chronic Hepatitis

Woodchucks infected with WHV display pattern of liver disease and age-dependent rates of development of CH similar to those in human HBV infection. In search for indicators capable of predicting in advance whether hepadnaviral infection will resolve or progress to CH, liver biopsies collected during AH from adult animals which finally either resolved AH or developed CH after administration of the same dose of WHV inoculum were investigated (16). The dynamics of intrahepatic expression of selected cytokines, liver T cell influx, histological severity of hepatitis, and serum and hepatic WHV loads were assessed. The data revealed that recovery from AH was characterized by a significantly greater hepatic expression of IFN-γ and CD3, an indicator of T cell infiltration, an increased transcription of TNF-α, a greater histological severity of liver inflammation, and by lower hepatic loads of WHV than those detected in animals in which AH advanced to CH. For instance, the estimated liver load of WHV during AH was 2.7-fold lower for animals that resolved AH than for these which established CH. The study also revealed that the elevated hepatic transcription of IFN-γ, TNF-α, and CD3 endured for years after resolution of AH. This was in agreement with the findings of residual WHV replication and minimal intermittent liver inflammation continuing normally for life after seemingly complete resolution of AH (29). Evaluation of similar parameters in liver biopsies acquired from healthy woodchucks in which the outcome of WHV AH was known did not demonstrate such predictive value. Overall, the study documented that the development of CH can be predicted well in advance by analyzing liver tissue in the acute phase of infection.

The above study was completed in the adult animals in which AH either subsided or advanced to CH in the setting of the mature immune system. It is note that woodchucks infected as adults with liver pathogenic doses of WHV develop CH at the rate not >25–30%. This is in contrast to WHV infection acquired in the neonatal period, which likely due to the immunological immaturity of the host, establishes CH in the majority of neonates. This somewhat resembles a situation in children born to mothers chronically infected with HBV or those exposed to virus very early in life which tend to develop much high rates of CH than adults if untreated. Despite significant immunological differences between adults and neonates, the parameters typifying the early phase of WHV infection progressing to recovery or CH identified in the adult woodchucks were closely comparable to those reported for neonatal infection (159, 160). Hence, recovery from AH in the neonatal period coincided with greater levels of hepatic IFN-γ and TNF-α transcription, augmented severity of hepatitis, and with lower hepatic loads of WHV. Taken together, despite significantly different frequencies of spontaneous recovery or progression to chronic disease in adult and neonatal animals, the same liver parameters measured in the acute phase of infection predict outcome of hepatitis.

## Toll-Like Receptor 1–10 Expression in Different Forms of WHV Infection and Stages of Experimental Hepatitis

Toll-like receptors (TLRs) are important mediators of immune responses which contribute to immune recognition of microbes and to the immunopathogenesis of diseases and cancers in general. There is now a wealth of data on the TLRs' expression, their functions and roles in a variety of diseases. A significant progress has also been made in recognition of TLRs' role in the pathogenesis of hepatitis B and in therapeutic potential of their antagonists in HBV infection (25). The woodchuck model plays a particularly important role in these studies. As an example, a potent agonist of TLR-7 (GS-9620) has been developed and tested in chimpanzees and woodchucks, and its evaluation has advanced to clinical trials. The results indicate that both innate and adaptive immunity contribute to sucessful antiviral response (161–164). However, the response was not uniformly effective among animals and patients treated. Recently, a new TLR-7 activating compound (APR002), designed to be preferentially delivered to the liver, was investigated in WHV-infected woodchucks in comination with ETV (165). The virological and immunological equivalence of functional HBV cure was achieved in some animals which appeared to be due to immunological modulation rather then augmented antiviral efficacy of the tretment (166). In another recent study, agonist of TLR-8 (GS-9688) has shown ability of sustained inhibtion of WHV replication in some chronically infected woodchucks when administered alone, as evidenced by decline in serum WHsAg and hepatic WHV cccDNA to levels undetectable by the assays employed (22). In this context, a variety of factors among which individual discrepancies in the TLRs' expression and cooperation between TLRs and functionally important downstream molecules, and individual differences in the kinetics of virus infection and in the degree of inflicted inflammation and liver damage could play a role.

Considering the above, comprehensive recognition of the TLR expression profiles in sequential stages of WHV infection and hepatitis was recently completed (167). The assessment of the transcription profiles of woodchuck TLRs 1 to 10 in serial liver biopsy and PBMC samples from the pre-infection and pre-acute periods to AH followed by SLAH and SOI or CH, and from animals with POI was made. This was supplemented by analysis of the TLR transcription profiles in whole livers and in primary hepatocytes isolated from these livers. This last comparison was important to determine how TLRs' expression differs between hepatocytes, which are the principal site of virus replication, and the liver where TLRs' transcription could be influenced by cells of inflammatory infiltrations, sinusoidal lining and by blood cells within hepatic vasculature. Since there are not yet specific antibodies against the majority of woodchuck TLRs and molecules facilitating their downstream effects, quantitative TLRs' gene expression analysis provided the most direct approach to recognize TLR characteristics of different stages of WHV infection and associated liver disease. The study generated an abundance of data among which the most interesting showed that liver biopsies from AH and CH demonstrated significantly enhanced transcription of the majority of TLRs when compared to healthy woodchucks and animals with other stages of infection. Also, in contrast to whole liver tissue, hepatocytes from CH displayed significantly lower transcription or a trend toward suppression of several TLRs when compared to hepatocytes from healthy woodchucks and animals with other forms of infection or hepatitis. This implied that hepatocyte innate immune response is in fact downregulated in the course of CH. In contrast to hepatocytes, upregulated expression of some TLRs characterized PBMC during CH. Overall, the study indicated that: (1) TLRs' expression widely varied between different forms of infection and to a significant degree depended on whether infection was accompanied or not by hepatitis; (2) Analysis of the TLRs' transcription in hepatocytes provided more distinct association with form of infection or stage of hepatitis than analysis of total liver tissue. This was best exemplified in CH and SOI continuing after SLAH, and (3) TLRs' expression profiles in PBMC overall poorly mirrored those in livers and hepatocytes from infected animals. The study also delineated the profiles of TLRs 1–10 expression during sequential stages infection in individual animals from the healthy state to AH/SLAH/SOI or AH/CH and the baseline TLRs' expression in healthy woodchucks.

Potential applications of TLRs in therapy of CH type B which were examined in the woodchuck model were reviewed previously (12, 25, 168). Among others, it was shown that activation of TLR-2-dependent innate immune response was associated with a decreased replication of WHV in primary woodchuck hepatocytes and HBV in HBV-transcribing HepG2.2.15 cells (169). This coincided with the activation of NF-κB and cell MAPK/ERK and PI2K/Akt signaling pathways, as well as with production of pro-inflammatory cytokines by woodchuck hepatocytes. WHV infection modulated TLR-2 expression on PBMC and this reversely correlated with WHV DNA levels in woodchucks with AH and in animals with CH treated with ETV. In related study, TLR-2 agonists transiently enhanced production of IL-6 and TNF-α, increased CD8+ T cell response, and augmented clearance of HBV in the hydrodynamic injection model of HBV infection (170).

## Concluding Remarks

The woodchuck model of hepatitis B has significantly contributed to the recognition of the natural history of hepadnaviral infection and the biological and pathogenic properties of hepadnaviruses, and to identification and characterization of occult HBV infection and its pathological outcomes. Reproducible experimental systems to investigate primary occult infection persisting indefinitely as a serologically and clinically silent but molecularly evident infection and secondary occult infection that continues in the presence virus-specific antibodies after resolution of a symptomatic infection were established. The woodchuck model significantly contributed to the identification of the pivotal, although not yet fully appreciated, role of the immune system as a reservoir of biologically competent hepadnavirus, even when the liver is seemingly not involved. The model assisted in the discovery of an extensive range of molecular and immunological mechanisms underlying development of liver disease of which some should be further explored, but many others await discovery. The uniqueness of the model relies on the close virological and pathogenic similarity to a human disease situation and on the unaltered environment in which interactions between virus and host occur. As such, the woodchuck-WHV infection should remain the standard for the discovery and validation of new pathogenic mechanisms and for preclinical drug testing.

## Author Contributions

TM conceived, designed, and wrote the manuscript.

## Conflict of Interest

The author declares that the research was conducted in the absence of any commercial or financial relationships that could be construed as a potential conflict of interest.
